# Acute Promyelocytic Leukemia: Pathophysiology, Diagnosis and Clinical Management

**DOI:** 10.3390/hematolrep17060066

**Published:** 2025-11-28

**Authors:** Meryeme Abddaoui, Youssef Aghlallou, Imane Tlemçani, Moncef Amrani Hassani

**Affiliations:** 1 Laboratory of Medical Sciences and Translational Research, Faculty of Medicine, Pharmacy and Dental Medicine of Fez, Sidi Mohammed Ben Abdellah University, Fès 30070, Morocco; 2 Institute of Cancer Research, Fès 30000, Morocco; youssefaghlallou@gmail.com; 3 Department of Hematology, Faculty of Medicine, Pharmacy and Dental Medicine of Fez, Fès 30070, Morocco; tlamcanimane@gmail.com (I.T.); moncef34@hotmail.com (M.A.H.)

**Keywords:** acute promyelocytic leukemia, *PML::RARA* fusion, coagulopathy, all-trans retinoic acid, arsenic trioxide, minimal residual disease

## Abstract

**Background/Objectives**: Acute promyelocytic leukemia (APL) is a distinct subtype of acute myeloid leukemia characterized by the t(15;17)(q24;q21) translocation, generating the *PML::RARA* fusion gene that blocks myeloid differentiation and drives leukemogenesis. Despite advances in therapy, early mortality remains a major challenge due to severe coagulopathy. This review aims to summarize recent insights into APL pathophysiology, diagnostic approaches, and management strategies. **Methods:** We performed a comprehensive review of the literature addressing the molecular mechanisms of APL, its associated coagulopathy, and current diagnostic and therapeutic standards, with a focus on evidence-based recommendations for clinical practice. **Results:** The hallmark PML: RARA oncoprotein disrupts nuclear body function and retinoic acid signaling, resulting in differentiation arrest and apoptosis resistance. APL-associated coagulopathy arises from overexpression of tissue factor, release of cancer procoagulant, inflammatory cytokines, and annexin II-mediated hyperfibrinolysis. Diagnosis requires integration of cytomorphology, immunophenotyping, coagulation studies, and molecular confirmation. Immediate initiation of all-trans-retinoic acid (ATRA) upon clinical suspicion, combined with aggressive supportive care, is critical to control bleeding risk. **Conclusions:** APL is now a highly curable leukemia when recognized early and treated with targeted therapy. Rapid diagnosis, prompt ATRA administration, and meticulous hemostatic support are essential to reduce early mortality. Further refinements in minimal residual disease monitoring are expected to improve patient outcomes.

## 1. Introduction

Acute promyelocytic leukemia (APL) represents between 5 and 20% of adult acute myeloid leukemia cases, with an estimated annual incidence of 1 to 7.4 cases per 1,000,000 person-years. It is characterized by a reciprocal and balanced translocation between the promyelocytic leukemia protein (*PML*) gene on chromosome 15 and the retinoic acid receptor α (*RARA*) gene on chromosome 17 [1,2]. The reciprocal t(15;17) translocation, found in more than 95% of APL cases, leads to the formation of the *PML::RARA* fusion protein, an oncogenic chimeric protein that plays a central role in leukemogenesis by interfering with multiple cellular pathways, resulting in increased proliferation of myeloid progenitors and a differentiation block at the promyelocytic stage [3,4]. In addition to the classical *PML::RARA* fusion, several rare *RARA* rearrangements have been described, including *PLZF::RARA*, *NPM1::RARA*, *STAT5B::RARA*, *PRKAR1A::RARA*, *BCOR::RARA*, and *FIP1L1::RARA*. Among these, *PLZF::RARA* [t(11;17)(q23;q21)] is the most frequent, accounting for approximately 1% of APL cases. *PLZF* functions as a transcriptional repressor involved in cell cycle regulation and myeloid differentiation. The biological behavior and treatment response of these atypical variants differ from those of classical APL, emphasizing the need for precise molecular characterization [4,5].

The onset of APL is usually associated with a severe thrombohemorrhagic diathesis, mainly driven by disseminated intravascular coagulation (DIC), which remains the leading cause of early mortality [6,7]. A systematic screening panel, that includes prothrombin time (PT), activated partial thromboplastin time (aPTT), D-dimers, and fibrinogen levels should be promptly implemented to aid in early diagnosis and guide the immediate initiation of supportive care aimed at preventing hemorrhagic complications [8,9,10]. Classical APL can be recognized by a distinct morphology of abnormal promyelocytes with a heavy granulation pattern and characteristic cells containing single Auer rods or bundles of Auer rods in the cytoplasm (faggot cells). Cytomorphology plays a crucial role in the early diagnostic orientation, as it often raises immediate suspicion of APL, and allows for prompt intervention. However, in certain cases, morphologic overlap with other subtypes of acute myeloid leukemia (AML) with reactive conditions may pose a diagnostic challenge, thereby necessitating additional investigations. Flow cytometry is a key complementary tool that confirms the myeloid origin of the blasts detected in peripheral blood or bone marrow aspirate. Most APL cases share a similar immunophenotype, negative for CD34 and HLA-DR, and positive for CD13, CD33, and CD117. MPO is strongly positive [11]. Of note, the immunophenotype described above is not specific to APL and can be seen in other types of AML. Ultimately, molecular confirmation through detection of the t(15;17)(q24;q21) translocation or the *PML::RARA* fusion transcript remains the definitive diagnostic criterion, enabling classification and initiation of targeted therapy [12]. The use of all-trans retinoic acid (ATRA) and arsenic trioxide (ATO) has revolutionized APL therapy, enabling exceptional cure rates and representing a paradigm shift away from the standard cytotoxic regimens employed in other malignancies [5]. This review aims to summarize recent insights into APL pathophysiology, particularly the mechanisms of coagulopathy, and to provide an updated overview of current diagnostic approaches, with a focus on APL cases harboring the *PML::RARA* fusion, as most clinical literature and data pertain to patients with this variant.

## 2. Pathophysiology of Acute Promyelocytic Leukemia

**Figure 1:**
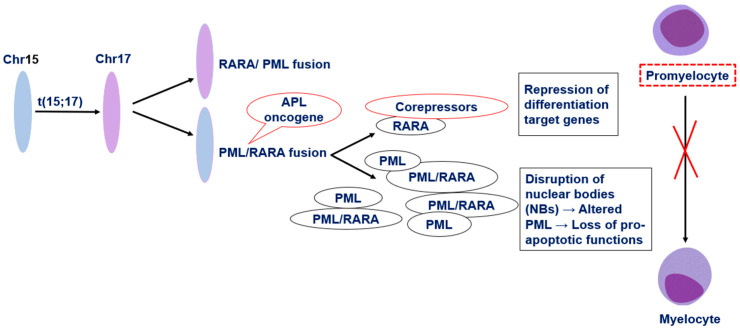
Schematic representation of chromosomal translocation t(15;17) that occurs at APL: The chromosomal translocation t(15;17) generates the PML/RARA fusion protein, which disrupts regulation of differentiation target genes and alters PML nuclear bodies, leading to a block in promyelocyte maturation [16,17].

Under physiological conditions, PML, also known as TRIM19, is a nuclear protein encoded by the PML gene on chromosome 15q24. It represents the structural core of promyelocytic leukemia nuclear bodies (PML-NBs), which are dynamic, membrane-less nuclear domains involved in the regulation of essential cellular processes. PML-NBs act as multifunctional platforms that recruit and concentrate various regulatory proteins, thereby modulating apoptosis, senescence, DNA damage response and repair, telomere homeostasis, and p53-mediated stress signaling [13,14,15]. RARA acts as a ligand-dependent transcription factor that heterodimerizes with retinoid X receptors (RXR) to regulate genes involved in self-renewal and differentiation. Alongside the tumor-suppressive functions of PML, this coordinated activity preserves genomic integrity and ensures proper hematopoietic differentiation [16]. However, *PML::RARA* disrupts PML nuclear bodies (PML-NBs), impairing the tumor-suppressive functions of PML and promoting cell survival. Mechanistically, the fusion protein prevents proper sumoylation-dependent assembly and phase separation of PML, thereby abolishing the structural integrity of PML-NBs [13]. In parallel, the RARA moiety of the fusion protein alters normal retinoic acid signaling. Unlike the wild-type RARA, which activates the transcription of differentiation-associated genes upon ligand binding. *PML::RARA* aberrantly recruits transcriptional co-repressors such as nuclear receptor corepressor (N-CoR), silencing mediator of retinoid and thyroid receptors (SMRT), and histone deacetylases (HDACs) to retinoic acid response elements (RAREs), resulting in sustained transcriptional repression [16]. This silencing of myeloid differentiation programs causes a maturation arrest at the promyelocyte stage, leading to the pathological accumulation of immature blasts in the bone marrow (Figure 1).

## 3. Mechanisms of Coagulopathy in APL

Following vascular injury, exposure of tissue factor (TF) initiates the extrinsic pathway by binding factor VIIa, leading to activation of factor X. Together with factor V, it forms the prothrombinase complex that generates thrombin. Thrombin acts as the key effector of coagulation: it converts fibrinogen into fibrin, activates factors V, VIII, and XI, and stimulates platelet activation and aggregation, creating a procoagulant surface that stabilizes clot formation. To avoid excessive thrombosis, several anticoagulant pathways maintain balance. Thrombomodulin–thrombin complexes activate protein C, which with protein S inactivates factors Va and VIIIa, while antithrombin III neutralizes thrombin and factor Xa. In parallel, the fibrinolytic system, mediated by tissue plasminogen activator (tPA) and urokinase (uPA), produces plasmin to degrade fibrin, a process tightly regulated by inhibitors such as α2-antiplasmin and Thrombin-Activatable Fibrinolysis Inhibitor (TAFI) [18,19].

**Figure 2:**
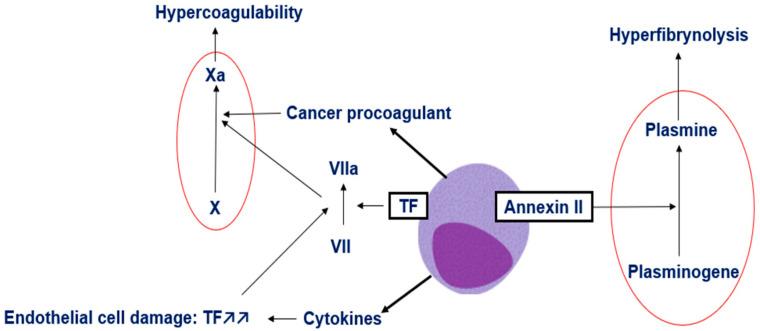
Pathophysiology of APL-associated coagulopathy, illustrating the mechanisms leading to bleeding and thrombotic complications in patients with APL [22].

In APL, leukemic promyeloblasts exhibit markedly elevated expression of TF, primarily driven by aberrant activation of the TF promoter via the dysregulated RARα signaling pathway [20]. This overexpression of TF initiates the extrinsic coagulation cascade promoting coagulation abnormalities in APL. In addition to this TF-driven mechanism, another significant contributor to the hypercoagulable state in APL is cancer procoagulant, a cysteine protease released by tumor cells that can directly activate factor X independently of factor VII [7]. Furthermore, TF-containing microparticles (TFMPs), which are membrane vesicles shed from leukemic promyeloblasts, have been identified in APL. These microparticles express functionally active TF and are thought to amplify thrombin generation and contribute to the systemic coagulopathy observed in APL, positioning them as additional key mediators of the prothrombotic phenotype [21]. A final etiology underlying the prothrombotic state in APL lies in the release of inflammatory cytokines by malignant cells, which damage the vasculature and activate multiple components of the coagulation cascade. Elevated levels of IL-1β, IL-6, and TNFα in patients with AML and APL further enhance endothelial tissue factor expression, while TNFα suppresses thrombomodulin transcription, promoting hypercoagulability and dysregulation of coagulation. The combined effect of tissue factor, cancer procoagulant, and proinflammatory cytokines from malignant promyelocytes results in excessive thrombin generation, leading to consumptive coagulopathy and increasing the risk of both thrombosis and hemorrhage (Figure 2) [7].

Fibrinolysis play a significant role in the coagulopathy associated with APL [7]. Overexpression of annexin II, a surface receptor and activator of tPA and uPA on APL promyeloblasts, contributes to excessive fibrinolysis and is a major factor in hemorrhage [7,22]. In addition, APL is characterized by markedly reduced levels of alpha2-antiplasmin, impairing the ability to counteract the disease-driven increase in plasmin generation and activity [9]. Furthermore, several studies have demonstrated that PAI-1 activity and the formation of tPA/PAI-1 complexes are substantially reduced in APL patients, likely as a result of proteolytic degradation of PAI-1, which prevents adequate control of disease-driven tPA hyperactivity [9]. A final mechanism contributing to hemorrhage in APL is impaired primary hemostasis [9,22]. Most patients present with thrombocytopenia at diagnosis, which predisposes them to early bleeding events.

## 4. Acute Promyelocytic Leukemia Diagnosis

### 4.1. Clinical and Morphological Assessment

APL is both a diagnostic and therapeutic emergency, as patients consistently present with a clinical picture that includes coagulopathy leading to a significant risk of bleeding. A complete blood count (CBC) is usually the first step, typically revealing pancytopenia [23]. Anemia and thrombocytopenia are common, and if hemoglobin or platelet levels are dangerously low, immediate transfusions are indicated. Some cases instead present with leukocytosis, a factor that plays a critical role in risk-adapted treatment decisions [5]. Bone marrow examination remains central to the morphological diagnosis. Abnormal promyelocytes usually account for ≥10% of marrow cells, often displaying eccentric and bilobed nuclei, folded contours, and prominent nucleoli [24,25]. Their cytoplasm contains irregular azurophilic granules and characteristic Auer rods, which may be seen in bundles, forming the so-called faggot cells, a hallmark of the hypergranular variant of APL [26]. In contrast, the hypogranular or microgranular variant shows a relative paucity or absence of visible cytoplasmic granules, along with bilobed or reniform nuclei [27]. This variant is frequently associated with higher leukocyte counts and may pose diagnostic challenges [28]. Rare morphological subtypes, including basophilic or atypical forms, have also been described and require careful recognition to avoid misclassification.

### 4.2. Immunophenotyping by Flow Cytometry

While the distinctive morphology of APL often supports its identification, exclusive dependence on morphological assessment can be inadequate in cases with unusual or atypical features. Flow cytometry immunophenotypic analysis is a routinely used tool and an indispensable component in the diagnosis, classification, prognostication, and monitoring AML subtypes, including APL. It identifies marker profiles with high diagnostic value for APL by combining positive and negative marker expression to support diagnosis. This analysis is performed on fresh bone marrow samples, which typically exhibit an immunophenotype characterized by high side scatter (SSC) and positivity for CD13, CD33, CD117, CD64, and bright MPO, along with double negative of CD34, HLA-DR [11,29]. The microgranular variant of APL usually shares a similar immunophenotype with the hypergranular form, except for the expression of CD34 [30]. Expert evaluation of this immunophenotypic pattern has been shown to effectively support the diagnosis of APL at the molecular level. Evaluation of measurable residual disease (MRD) in patients with APL can be optimized by combining the classic Leukemia-Associated Immunophenotype (LAIP) approach with the Different-from-Normal (DfN) strategy. This approach utilizes a panel of antibodies targeting CD9, CD117, CD123, CD11b, CD15, CD33, CD45, CD56, CD64, and HLA-DR to specifically profile malignant promyelocytic cells, enabling comprehensive tracking of all aberrancies, including those emerging after initial diagnosis [31]. However, prospective data on the immunophenotypes of residual leukemic cells, whether in recovering patients, MRD-positive cases, or relapsed APL, remain limited. In this context, flow cytometry retains a primarily complementary role, particularly for diagnostic confirmation and identification of aberrant profiles at diagnosis, while systematic MRD monitoring relies mainly on molecular methods targeting the *PML::RARA* fusion [31].

### 4.3. Coagulation Studies

Evaluation of coagulation parameters is essential in the diagnosis and management of APL, which is frequently complicated by severe, early-onset coagulopathy. This hemostatic disturbance is primarily driven by aberrant expression of TF and annexin II on promyelocytic blasts, leading to activation of the extrinsic coagulation pathway and excessive plasmin generation. Together, these mechanisms create a highly unstable balance between bleeding and thrombosis. Routine laboratory evaluation should therefore include a full coagulation profile at baseline and serially during induction therapy. Prolongation of PT is common, reflecting depletion of extrinsic and common pathway factors (VII, X, V, II, and fibrinogen). Simultaneous activation and consumption of intrinsic pathway factors (VIII, IX, XI) result in prolonged aPTT. Low fibrinogen levels (<150 mg/dL) and markedly elevated D-dimer concentrations are characteristic of the disseminated intravascular coagulation (DIC)-like state seen in APL [32]. Recent guidelines emphasize the D-dimer/fibrinogen ratio as an additional prognostic tool, with higher ratios correlating with increased bleeding risk [7]. Thrombocytopenia further exacerbates the hemorrhagic diathesis, underscoring the importance of early and aggressive supportive care.

### 4.4. Molecular and Cytogenetic Analyses

Molecular genetic confirmation of the *PML::RARA* translocation should be obtained as rapidly as possible to enable prompt initiation of induction therapy. The detection of the APL-specific genetic lesion can initially be performed using conventional karyotyping, which frequently identifies the balanced translocation t(15;17)(q24.1;q21.2), corresponding to the molecular fusion of the *PML* and *RARA* genes. Fluorescence in situ hybridization (FISH) targeting the *PML::RARA* rearrangement is particularly valuable when urgent diagnostic confirmation is required (<24 h) or when conventional karyotyping is suboptimal, for example, due to poor chromosome morphology or insufficient cell growth, which may result in cryptic or undetectable translocations [33]. However, these cytogenetic methods do not provide information on the exact fusion breakpoint and a negative result does not exclude the diagnosis, especially in cases involving atypical or cryptic *PML::RARA* rearrangements. In this context, reverse transcription polymerase chain reaction (RT-PCR) is essential and widely used for the detection of the *PML::RARA* fusion transcript. In practice, RNA extracted from bone marrow or peripheral blood is reverse transcribed into cDNA, followed by conventional RT-PCR, enabling amplification and identification of the transcript. The specific fusion isoforms, corresponding to the breakpoints *bcr1* (long form, L), *bcr2* (variant form, V), and *bcr3* (short form, S), allow precise confirmation of the t(15;17) translocation in APL. While RT-PCR efficiently detects typical *PML::RARA* transcripts, sporadic cases may present atypical isoforms that are not recognized by conventional primers, requiring customized assays or sequencing to identify the fusion [34,35]. 

Emerging technologies, such as optical genome mapping (OGM) or next-generation-sequencing (NGS) based panels, offer comprehensive genome-wide detection of structural variants and gene fusions, including cryptic or complex rearrangements, and may further improve diagnostic accuracy in APL, although their use in routine urgent diagnosis is still limited [36]. 

Finally, it is essential to highlight that reverse transcription polymerase chain reaction (RT-PCR) remains the gold standard [37]. This method enables the precise identification of specific *RARA* rearrangements and is indispensable for diagnosis, treatment monitoring, and MRD assessment.

## 5. Clinical Management

A provisional diagnosis of APL based on clinical presentation and hematologic assessment is routinely available before genetic confirmation, to enable prompt initiation of supportive care. Immediate ATRA treatment which targets the Retinoic acid (RA) receptor and induces terminal differentiation of APL blasts, is critical as it is known to rapidly reduce the biologic drivers of APL-associated coagulopathy and does not have a deleterious effect in treating other AML subtypes. Supportive transfusion therapy is central to the management of APL, particularly in the early phase when life-threatening coagulopathy predominates. According to the 2022 European LeukemiaNet (ELN) guidelines, cryoprecipitate or plasma transfusions are recommended to correct hypofibrinogenemia, while platelet transfusions are indicated to counteract severe thrombocytopenia [33,38]. Both fibrinogen and platelets are essential for the initiation and propagation of clot formation; their deficiency significantly amplifies the risk of catastrophic hemorrhage, which remains the major cause of early mortality in APL. The recommended transfusion thresholds are to maintain fibrinogen concentrations above 100–150 mg/dL and platelet counts above 30,000–50,000/µL [7,35]. Close laboratory monitoring every six hours, including platelet counts, fibrinogen, PT/INR, and aPTT, is recommended to promptly detect and manage consumptive coagulopathy [7]. Pharmacologic approaches such as heparin and recombinant thrombomodulin (rTM) have been investigated for their potential to modulate coagulation, heparin through antithrombin III mediated inhibition of factors II and X, and rTM through protein C activation and annexin II downregulation. However, their routine use is not recommended due to the risk of hemorrhage and limited clinical benefit, and antifibrinolytic therapy is also discouraged outside clinical trials in the absence of robust randomized evidence. Beyond pharmacologic interventions, invasive procedures during induction should ideally be deferred until coagulation parameters have stabilized.

The white blood cell (WBC) count at presentation is a key prognostic factor that stratifies patients into low- or intermediate-risk (standard-risk) and high-risk categories. Low- or intermediate-risk APL is defined by a WBC count ≤ 10 × 10^9^/L, while a WBC count > 10 × 10^9^/L identifies high-risk APL. In low- or intermediate-risk patients, ATRA combined with ATO without chemotherapy is now established as the standard of care, providing highly effective outcomes. This regimen eradicates leukemic promyelocytes through synergistic induction of differentiation and apoptosis, leading to morphological and molecular remission while avoiding the cardiac and hematologic toxicities associated with anthracyclines [5,10,35,39,40].

In high-risk APL patients with significantly elevated WBC counts, induction with ATRA and ATO alone can accelerate the rapid proliferation of leukocytes, increasing the risk of early differentiation syndrome (DS) and rapid clinical deterioration [41,42]. To address this, two main treatment strategies have been evaluated for high-risk patients: ATRA, ATO, and cytoreductive therapy; or ATRA combined with conventional chemotherapy. Both approaches have demonstrated efficacy and may be selected based on drug availability and patient-specific considerations [33]. The clinical question remains whether high-risk patients can be induced with ATRA and ATO without chemotherapy. A large randomized trial involving most European cooperative groups (APOLLO study, NCT02688140) was conducted to compare the efficacy of ATO in combination with ATRA plus low-dose idarubicin versus the standard ATRA plus anthracycline-based chemotherapy regimen in patients with high-risk APL. The results of the trial support the use of ATO and ATRA, with low-dose idarubicin, for the treatment of newly diagnosed high-risk APL patients, as this combination demonstrates superior efficacy and tolerability compared with conventional chemotherapy [43]. Despite the proven success of ATRA–ATO in standard-risk disease, there is still no contemporary evidence supporting its use as a standalone regimen in high-risk patients. Although ATRA plus ATO with an anthracycline remains the preferred induction regimen for high-risk patients at most centers, gemtuzumab ozogamicin (GO), a CD33 targeted antibody-drug conjugate, represents a valid alternative for patients ineligible for anthracycline therapy due to cardiac or other comorbidities, as recommended in the current NCCN guidelines [5,43,44,45]. It should also be noted that the same ATRA–ATO–based treatment protocols used for classical PML::RARA–positive APL are not applicable to APL variants, which usually require intensive chemotherapy and novel targeted therapies guided by molecular diagnostics [33].

Following induction therapy, consolidation represents a pivotal step to eradicate residual leukemic clones and ensure sustained remission. In low- and intermediate-risk APL, consolidation with ATRA and ATO after ATRA/ATO-based induction has yielded excellent long-term outcomes, achieving durable molecular remission without the need for cytotoxic chemotherapy. In patients treated with conventional ATRA plus chemotherapy regimens, the inclusion of ATRA during consolidation confers an additional clinical benefit. For high-risk cases, those who have successfully achieved remission with ATRA and ATO combined with an anthracycline may undergo consolidation with ATRA and ATO alone, allowing omission of further chemotherapy [5,35].

Advances in ATRA/ATO-based induction and consolidation have challenged the traditional role of maintenance therapy in APL, particularly given the exceptionally low relapse rates observed in recent clinical trials. Patients treated with ATRA plus ATO received no maintenance therapy, and relapse after consolidation was exceedingly rare. However, maintenance therapy should be considered in high-risk patients who were treated with ATRA plus chemotherapy (without ATO) [33,46].

**Figure 3:**
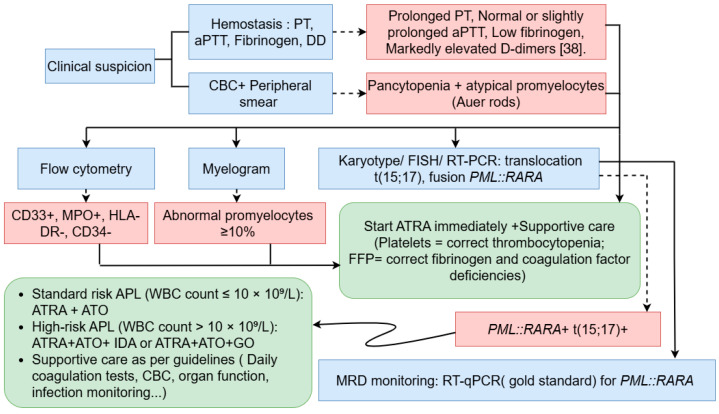
Diagnostic workflow and early management of APL, including laboratory evaluation, risk stratification, and initiation of induction therapy [7,10,11,25,38,43,45].

Although APL is associated with high cure rates, relapse can still occur in a fraction of adult patients after achieving complete remission (CR). MRD monitoring has therefore become a cornerstone of APL management, particularly in high-risk patients, as it enables early relapse prediction and guides preemptive therapeutic interventions. Molecular relapse, indicated by rising or persistent *PML::RARA* transcript levels, usually precedes overt hematological relapse, which is defined by the reappearance of Abnormal promyelocytes in a single bone marrow sample and should be confirmed through molecular testing. RT-qPCR targeting the *PML::RARA* transcript is widely used for MRD detection because of its high sensitivity, reproducibility, and standardization. Current guidelines recommend performing the first MRD assessment at the end of consolidation, as persistent positivity at this time strongly correlates with relapse risk. In high-risk patients, ongoing molecular monitoring every three months for 2–3 years is advised, whereas extended surveillance is generally unnecessary in low- and intermediate-risk patients who achieve molecular negativity, except in specific clinical situations (Figure 3) [20,47].

In cases of molecular or hematologic relapse, the choice of salvage therapy depends on the initial treatment regimen: ATRA + ATO is preferred after ATRA + chemotherapy, and vice versa. The goal is to achieve molecular remission prior to hematopoietic stem cell transplantation (HSCT). According to NCCN (2024) and ELN recommendations, autologous HSCT is indicated for MRD-negative disease, whereas allogeneic HSCT is reserved for MRD-positive cases. This sequential approach aligns with the modern therapeutic paradigm for relapsed APL and represents a potentially curative strategy [33,47,48].

## 6. Conclusions

Acute promyelocytic leukemia represents a distinct and highly curable subtype of acute myeloid leukemia when recognized promptly and treated appropriately. Advances in understanding its unique pathophysiology, driven by the *PML::RARA* fusion and its downstream disruption of myeloid differentiation, have led to targeted therapies that have dramatically improved patient outcomes. Nonetheless, early mortality remains a challenge, mainly due to life-threatening coagulopathy, underscoring the critical importance of rapid diagnosis, early identification of biological markers or scoring parameters to predict hemorrhagic and thrombotic risk, and the immediate initiation of ATRA-based therapy. The integration of modern hematologic, cytogenetic, and molecular tools enables precise and timely diagnosis, while advances in MRD monitoring continue to refine post-remission management strategies. This review primarily summarizes current diagnostic and therapeutic advances but is limited by the heterogeneity of available data, especially regarding real-world management and outcomes in resource-limited settings. Future research should focus on developing validated risk-scoring models that integrate clinical, hemostatic, and molecular parameters to better predict early death and relapse, standardizing MRD monitoring techniques and determining their optimal timing and thresholds for clinical decision-making, exploring novel targeted agents or combination strategies for relapsed or refractory cases, and improving access to ATO-based regimens and emergency diagnostic pathways to reduce early mortality worldwide. Continued multidisciplinary efforts to refine diagnostic algorithms, optimize early detection, and ensure equitable access to specialized care will be essential to sustain and extend the remarkable success achieved in APL treatment.

## Data Availability

No new data were created or analyzed in this study.
